# Fabrication of Poly(vinyl alcohol)-Polyaniline Nanofiber/Graphene Hydrogel for High-Performance Coin Cell Supercapacitor

**DOI:** 10.3390/polym12040928

**Published:** 2020-04-17

**Authors:** Hyeonseo Joo, Hoseong Han, Sunghun Cho

**Affiliations:** 1 School of Chemical Engineering, Yeungnam University, Gyeongsan 38541, Korea; hyeonseo.joo@dgist.ac.kr (H.J.); hoseong.han@student.unimelb.edu.au (H.H.); 2 Department of Energy Systems Engineering, DGIST, Daegu 42988, Korea; 3 Department of Chemical and Biomolecular Engineering, The University of Melbourne, Parkville, Victoria 3010, Australia

**Keywords:** polyaniline, nanofiber, hydrogel, multilayer graphene, supercapacitor, composite, conductive polymer

## Abstract

Electroactive polymer hydrogel offers several advantages for electrical devices, including straightforward synthesis, high conductivity, excellent redox behavior, structural robustness, and outstanding mechanical properties. Here, we report an efficient strategy for generating polyvinyl alcohol–polyaniline–multilayer graphene hydrogels (PVA–PANI–MLG HDGs) with excellent scalability and significantly improved mechanical, electrical, and electrochemical properties; the hydrogels were then utilized in coin cell supercapacitors. Production can proceed through the simple formation of boronate (–O–B–O–) bonds between PANI and PVA chains; strong intermolecular interactions between MLG, PANI, and PVA chains contribute to stronger and more rigid HDGs. We identified the optimal amount of PVA (5 wt.%) that produces a nanofiber-like PVA–PANI HDG with better charge transport properties than PANI HDGs produced by earlier approaches. The PVA–PANI–MLG HDG demonstrated superior tensile strength (8.10 MPa) and higher specific capacitance (498.9 F/cm^2^, 166.3 F/cm^3^, and 304.0 F/g) than PVA–PANI HDGs without MLG. The remarkable reliability of the PVA–PANI–MLG HDG was demonstrated by 92.6% retention after 3000 cycles of galvanostatic charge–discharge. The advantages of this HDG mean that a coin cell supercapacitor assembled using it is a promising energy storage device for mobile and miniaturized electronics.

## 1. Introduction

With the growing development of supercapacitor devices, there have been great improvements in electroactive polymer (EAP) nanomaterials with high specific capacitance [1,2,3]. Polyaniline (PANI) is one of the best EAPs for pseudocapacitors due to its straightforward synthesis, reversible doping/dedoping process, unique redox behavior, and high electrical conductivity [4,5,6,7,8,9]. Owing to their fascinating electrochemical and electrical properties, PANIs and derivatives can store huge amounts of electric charge through reversible oxidation–reduction reactions between positive and negative electrodes. Therefore, a number of studies on the synthesis of PANIs and derivatives for supercapacitor devices have been reported [4,5,6,7,8,9]. Despite their advantages, it is impossible to avoid volumetric degradation and scission of PANI chains when these chains are exposed to electrolyte ions during the charge/discharge process [4]. Accordingly, PANI-based supercapacitors usually suffer from limited cycle life and low reliability. As only PANI chains near the electrolyte ions participate in the charging/discharging process, the real capacitance of PANI is much lower than its hypothetical capacitance value [4]. To resolve these problems, it is necessary to develop a fabrication process that produces PANI with improved durability and reliability.

Recently, electroactive hydrogels (HDGs) have attracted interest as supercapacitor materials because of their high flexibility, durability, and remarkable structural stability during many charge/discharge cycles [5,6,10,11,12]. In addition, the three-dimensional polymer network structure of HDGs provides more surface area, allowing more electrolyte ions within the supercapacitor electrodes. Several studies on EAP HDGs, such as PANI [5,6], poly(3,4-ethylenedioxythiophene):poly(4-styrenesulfonate) [10], and polypyrrole [11,12], have evaluated them as supercapacitors. Li et al. reported a technique to manufacture a robust PANI HDG through a crosslinking reaction between boronic acid-functionalized PANI and polyvinyl alcohol (PVA) chains [5]. During this generation of PANI HDG, 3-aminophenyl boronic acid (3-ABA) played an important role, forming boronate (–O–B–O–) bonds between PANI and PVA chains. Despite the remarkable improvements in its tensile strength (5.3 MPa) and specific capacitance (306 mF/cm^2^) that resulted, PANI HDG needs further development to improve its electrochemical, electrical, and mechanical properties for use in high-performance supercapacitor devices. Especially, there have been few reports on the PVA content needed for the optimal performance of PANI HDG.

Graphene is a *sp*^2^-hybridized carbon nanomaterial having a one-atom-thick planar structure that is predicted to have a large specific surface area of 2640 m^2^/g [4,13]. The excellent electron mobility, flexibility, and thermal and mechanical properties resulting from this large surface area make graphene one of the most promising fillers for various EAPs [4,7,8,9,13,14]. The charge transport properties of PANI are remarkably enhanced through face-to-face interactions with graphenes, which has led to combining PANI and graphene sheets for high-performance supercapacitors [7,8,9]. In addition, graphene stores electric charge through rapid ion adsorption/desorption on its sheets, resulting in a faster charging time and increased capacitance of PANI–graphene composites [7,8,9]. However, there have been few reports on the synergistic effects of PANI and graphene on the supercapacitor performance of PANI HDGs. Thus, the development of high-performance PANI–graphene HDGs with excellent electrochemical, electrical, and mechanical performance remains a challenge.

Here, we report an efficient route to produce PVA–PANI–multilayer graphene (MLG) HDGs with excellent scalability and significantly improved mechanical, electrical, and electrochemical properties. We utilized our as-prepared PVA–PANI–MLG HDG for coin cell supercapacitors, which constitute a practical type of cell for evaluating the actual capacitive performance of the HDGs. PVA–PANI–MLG HDGs can be generated easily through the formation of –O–B–O– bonds between PANI and PVA chains. Strong intermolecular interactions between MLG, PANI, and PVA chains make PVA–PANI HDGs stronger and more robust than pristine PANI HDG. To investigate the effects of the PVA content on the morphological, electrical, and electrochemical properties of these HDGs, HDGs with different PVA contents were characterized using a field-emission scanning electron microscope (FE-SEM), cyclic voltammetry (CV), galvanostatic charge–discharge (GCD), cycling stability, and rate capability tests. Systematic studies on the effects of MLG on the electrical, electrochemical, and mechanical performance of the PVA–PANI–MLG HDGs were carried out using CV, GCD, electrochemical impedance spectroscopy (EIS), cycling stability, rate capability, and Ragone plots. The study culminated in the production of PVA–PANI–MLG HDG with excellent tensile strength (8.10 MPa), high specific capacitance (498.9 F/cm^2^, 166.3 F/cm^3^, and 304.0 F/g), and remarkable retention rate (92.6% retention after 3000 cycles of GCD cycles).

## 2. Materials and Methods

### 2.1. Materials

We purchased aniline (AN; 99%), ammonium persulfate (98%), PVA (99%, M_w_: 89,000–124,000), and 3-ABA from Sigma-Aldrich (St. Louis, MO, USA). Hydrogen chloride (35–37%) was obtained from Daejung Chemical & Metals Co., Ltd. (Siheung, Republic of Korea). We purchased MLG paste from MExplorer Co., Ltd. (Ansan, Republic of Korea); the average thickness and lateral size of graphenes in this material were approximately < 5 nm and 2–3 μm, respectively. The average aspect ratio of each graphene sheet in the MLG paste was approximately 1.1–2.0 [15]. In Raman spectroscopy, as we reported for our previous work, the intensity ratio of the D- to the G-band (*I*_D_/*I*_G_) of the MLG was about 0.39, whereas those of graphene oxide (GO) and rGO (reduced graphene oxide) are about 0.8–0.9 and 1.0–1.2, respectively [16,17]. This suggests that the MLG used in this work was exfoliated by an electrochemical route, unlike chemically converted graphenes. We purchased μm-thick polypropylene–polyethylene–polypropylene (PP–PE–PP) trilayer film from Celgard, LLC. (Charlotte, NC, USA). Coin cell assemblies (CR2032-type) including 0.1 mm-thick stainless steel foil and components were purchased from MTI Corporation (Richmond, CA, USA).

### 2.2. Fabrication of PVA–PANI–MLG HDG and Coin Cell Supercapacitors

**Figure 1:**
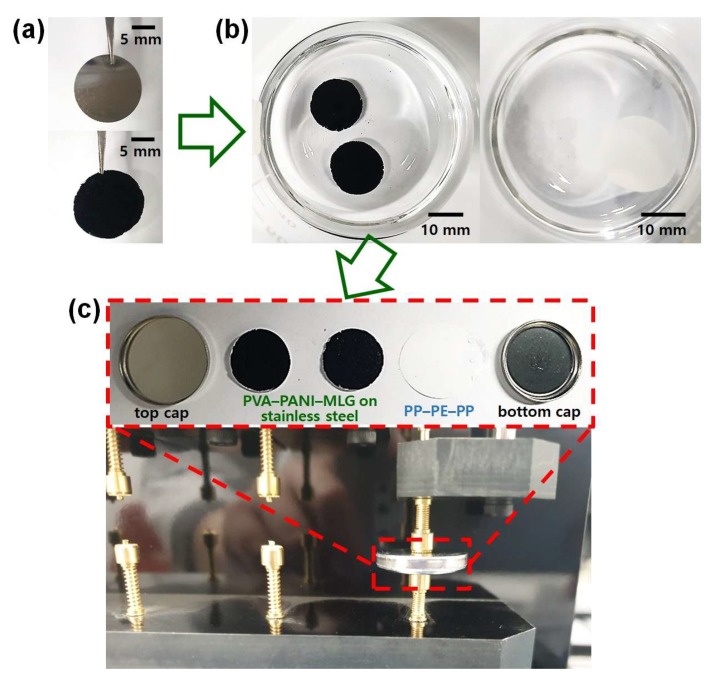
Overall procedures for electrode preparation and coin cell assembly steps: (**a**) digital images of stainless steel substrate (up) and PVA–PANI–MLG deposited on stainless steel substrate. (**b**) digital images PVA–PANI–MLG electrodes (left) and PP–PE–PP separator (right) immersed 1M H_2_SO_4_ aqueous solution. (**c**) Each component of a coin cell supercapacitor (up) and a coin cell mounted on cell jig (down).

In the first step, an oxidizing agent solution (solution A) was manufactured by dissolving 3 mmol of ammonium persulfate in 10 mL of deionized water. Ten mL of 5 M HCl solution were combined with 14.8 mmol of AN to form an aqueous anilinium ion solution (solution B). 1.46 mmol of 3-ABA and 60–300 mg of MLG paste were introduced to solution B and vigorously stirred for 0.5 h. Then, 30 mL of PVA solution (5–15 wt.% with respect to deionized water) were introduced to solution B, and the mixture was vigorously stirred for 0.5 h. To promote dispersions of each component in the reaction medium, solution B was placed in an ultrasonic bath (CPX2800H-E, Branson Ultrasonics Co., Danbury, CT, USA). The sonication of 0.5 h was performed at a power of 110 W and a frequency of 40 kHz. Solution A was added dropwise to the mixture of PVA and solution B, and the new mixture was vigorously stirred for 1 h. Every HDG polymerization step was carried out at 3 °C. These experimental steps produced a rigid and robust PVA–PANI–MLG HDG of 12.5 cm in diameter and 8.8 cm in height. The size of the PVA–PANI–MLG HDG depended on the amounts of reactants used (Table 1). The as-prepared HDGs were spread onto stainless steel foil substrates using a hydraulic pressing machine (HP, Ilsin Autoclave Corporation, Daejeon, Republic of Korea). The HDGs attached to steel substrate were pressed into 3 μm-thick thin films, and these films were cut into circles (15-mm diameter) (Figure 1a). To make successful electrical contact between the HDGs and the outer caps, stainless steel foil was utilized as a current collector. The PP–PE–PP trilayer film was cut into circles of 17-mm diameter. To promote the adsorption of electrolyte molecules in the electrode and membrane, we immersed the HDG electrodes and PP–PE–PP film in 1 M H_2_SO_4_ aqueous solution for 3 h (Figure 1b). We then produced sandwich-type supercapacitor coin cells consisting of a CR2032 button cap, two HDG electrodes, a PP–PE–PP membrane, and a top cap (Figure 1c). Each component was placed sequentially in a hydraulic crimper (MSK-110, MTI Corporation, Richmond, CA, USA) and sealed at a pressure of 50 kg/cm^2^.

### 2.3. Characterization of PVA–PANI–MLG HDG and Coin Cell Supercapacitors

We used an FE-SEM (S-4800, HITACHI, LTD, Hitachi, Japan) to study the morphology of our HDGs. A universal testing machine (UTM, Instron-5543, Instron Co., Norwood, MA, USA) was used to evaluate the mechanical properties of the HDGs according to the American Society for Testing and Materials standard D638 [17,18]. The mechanical properties of the samples were assessed at a cross-head speed of 10 mm·min^−1^, a temperature of 25 °C, and a relative humidity (RH) of 30%. The chemical bonds of the HDGs were investigated using a Fourier transform infrared (FTIR) spectrometer (Frontier, PerkinElmer Inc., Waltham, MA, USA). The electrochemical characteristics of the assembled HDG samples were evaluated using an electrochemical workstation (ZIVE SP2, Wonatech, Seoul, Republic of Korea). CV curves of the coin cells were recorded in the voltage range 0–1.0 V at a scan rate of 20 mV·s^−1^. GCD experiments were conducted by applying the voltages from 0 to 1.0 V at current densities from 0.30 to 7.20 mA/cm^2^. In the following data, the symbols *I*, Δ*t*, Δ*V*, *W*, *l*, and m represent the current, discharge time, voltage, electrode area, electrode volume, and electrode mass, respectively. The areal capacitances, *C_A_*, with units mF/cm^2^, of the HDG samples were determined using the equation *C_A_* = *I*Δ*t*/*W*Δ*V* [1,2,4,5,6,7,8,9,10,11,12,19,20,21]; the total *W* of the electrodes was measured to be 3.53 cm^2^. The volumetric capacitance *C_l_*, with units F/cm^3^, was calculated as *C_l_* = *I*Δ*t*/*l*Δ*V* [1,2,4,5,6,7,8,9,10,11,12,19,20,21]; the total *l* of the electrodes was measured to be 1.06 × 10^−2^ cm^3^. The gravimetric capacitance, *C_m_*, with units F/g, was calculated as *C_m_* = *I*Δ*t*/*m*Δ*V* [1,2,4,5,6,7,8,9,10,11,12,19,20,21]; the total *m* of the electrodes was fixed at 6 mg. The energy density, *E*, with units Wh/kg, was calculated as *E* = *C_m_*Δ*V^2^*/2, where Δ*V* is the voltage drop upon discharge [1,2,4,5,6,7,8,9,10,11,12,19,20,21]. The power density, *P*, with units W/cm^3^, was calculated as *P* = *E*/*t* [1,2,4,5,6,7,8,9,10,11,12,19,20,21]. The electrochemical impedance spectroscopy (EIS) characteristics of the HDG samples were recorded in the frequency range 1–10 MHz.

## 3. Results

**Figure 2:**
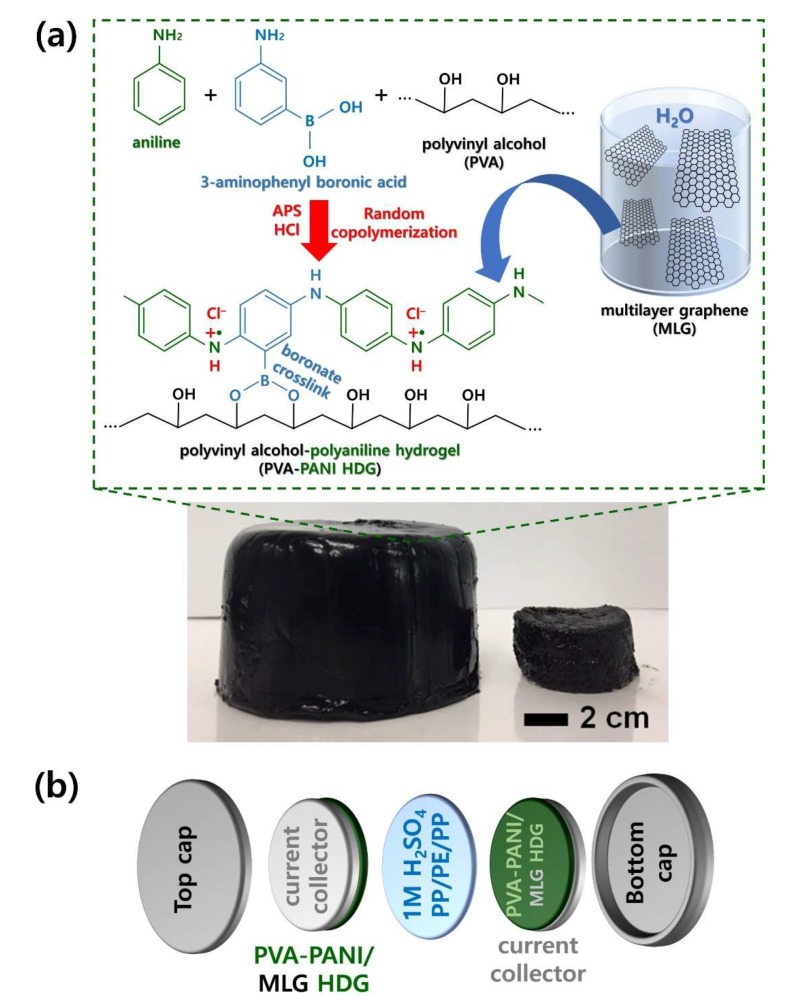
(**a**) Overall synthetic route for producing PVA–PANI–MLG HDG with tunable scalability. (**b**) Schematic illustration for a coin cell supercapacitor assembled with PVA–PANI–MLG HDG as electrode materials.

Figure 2 represents the overall fabrication procedure of PVA–PANI–MLG HDG for use in two-electrode symmetric supercapacitors. In the first step, random copolymerization of AN and 3-ABA monomers was carried out (Figure 2a), resulting in the generation of PANI chains with hydroxyl (–OH) groups. As two hydroxyl (–OH) groups are included in each repeating unit of PANI chains, it is possible to carry out condensation reactions between PANI and PVA chains. These reactions eliminated two water molecules per repeating unit, and formed –O–B–O– crosslinks between the PANI and PVA chains [5,6]. The crosslinks play an important role in creating the three-dimensional HDG structure. During the condensation reactions, MLG was embedded within the HDG. As the MLG used in our work was exfoliated by an electrochemical method, it suffers less from aggregation of sheets than conventional reduced graphene oxides [4]. In addition to the –O–B–O– crosslinks within the PVA–PANI HDG, the presence of MLG intensifies London dispersion, dipole–dipole, hydrogen bonding, and π–π stacking forces between the MLG, PVA, and PANI chains [5,9,22,23]. Hydrogen bonds occur between the –NH_2_ groups of PANI and –OH groups of PVA chains, and the –O–B–O– crosslinks can generate additional hydrogen bonds with both. In addition, the B atoms of the –O–B–O– crosslinks can form dipole–dipole forces with the H atoms of both of these groups [5,22,23]. As the graphene sheets contain *sp*^2^-hybridized carbon atoms, they can create π–π stacking with the PANI chains [9]. Due to the enhanced intermolecular interactions, the PVA–PANI–MLG HDG is more robust and durable than unmodified PVA–PANI HDG. As shown in the digital images of our HDGs, the PVA–PANI–MLG HDGs can easily be scaled; the maximum diameter of a PVA–PANI–MLG HDG obtained from our work was ~12.5 cm, which is ~2.5 times larger than that of HDG prepared by conventional methods (5 cm). This reaffirms that MLG enhances intermolecular forces within HDG, and the improved intermolecular strength of the resulting HDG supports the stable formation of PVA–PANI HDG [7,8,9]. This high scalability greatly increases the cost-effectiveness and process efficiency of producing HDGs for supercapacitors. As-prepared PVA–PANI–MLG HDG was utilized as electrode material in symmetric supercapacitors (Figure 2b). We manufactured the symmetric supercapacitors using a coin cell structure, which ensures their stability and practical applicability [19,20]. MLG has excellent mechanical properties that prevent the swelling and degradation of polymer chains when exposed to electrolyte ions, resulting in improved lifetime and reliability of the supercapacitors [7,8,9]. Furthermore, the electric charge stored at the interface between the MLG and electrolyte ions can further enhance the total capacitance of PVA–PANI HDGs, which store energy via redox reactions [7,8,9]. Therefore, we expect that coin cells employing PVA–PANI–MLG HDGs will provide superior supercapacitor performance compared to a PVA–PANI HDG sample.

**Figure 3:**
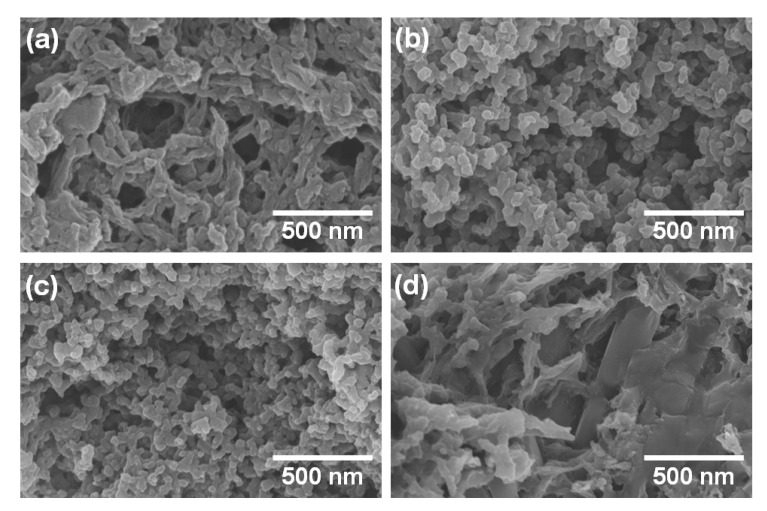
FE-SEM images of PVA–PANI HDGs with (**a**) 5 wt.% PVA, (**b**) 10 wt.% PVA, and (**c**) 15 wt.% PVA. (**d**) FE-SEM image of PVA–PANI–MLG HDG with 5 wt.% PVA and 3 wt.% MLG.

To identify the effects of PVA on the morphology of PANI HDGs, FE-SEM images of PVA–PANI HDGs with different PVA contents are shown in Figure 3a–c. With increasing amounts of PVA matrix, the lengths and sizes of the PANI nanomaterials became shorter and smaller. In particular, when the PVA content was 5 wt.%, nanofibers of 40–50 nm in diameter and 500–1000 nm in length were found within the PVA–PANI HDG, similar to the dimensions of pure PANI nanofibers (Figure 3a). PANI nanoparticles were also generated using PVA contents of 10 and 15 wt.%; the diameters of PANI nanoparticles found in these samples were about 40–60 and 30–40 nm, respectively (Figure 3b,c). These results indicate that the PVA matrix not only participates in the crosslinking reaction with the PANI chains, but also acts as a tailoring agent for adjusting the shapes and sizes of the resulting PANI nanomaterials [21]. More importantly, a higher aspect ratio of PANI nanofibers provides enhanced conjugation paths for delocalizing more electrons, resulting in lower internal resistance (IR) of the electrode materials [21]. When the PVA concentration was below 5 wt.%, the formation of the HDGs was not successful. For this reason, 5 wt.% was the optimal PVA concentration for achieving both successful formation and better charge transport properties of PVA–PANI HDGs. The addition of 3 wt.% MLG to PVA–PANI HDG made using 5 wt.% PVA resulted in PANI nanofibers and the MLG combining well within the resulting HDG (Figure 3d). This suggests that the synergistic effects from PANI nanofibers, PVA chains, and MLG lead to superior electrical, electrochemical, and mechanical properties in the resulting PVA–PANI–MLG HDG. The roles of each component contributing to synergistic effects are as follows: (1) The hydrogen bonding force between the PVA chains promotes the formation of mechanically and thermally strong hydrogels. In addition, PVA improves the dispersibility of PANI nanofibers and MLG through strong hydrogen bonding with PANI nanofibers and dipole-dipole interaction with MLG [5,22,23]. (2) Excellent redox properties and high electrical activity of PANI maximize the function of the prepared PVA–PANI–MLG HDG as a pseudocapacitor [4,5,6,7,8,9,21,22]. (3) MLG not only provides additional energy storage by the electric double-layer capacitor (EDLC) mechanism, but also reinforces the thermal and mechanical properties of polymeric materials such as PANI and PVA, thereby increasing the reliability and lifetime of the PVA–PANI–MLG HDG [7,8,9].

**Figure 4:**
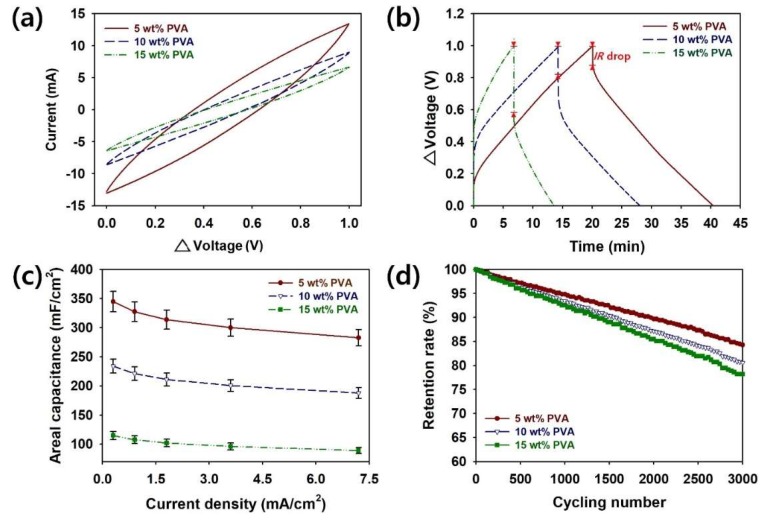
(**a**) CV curves, (**b**) GCD curves, (**c**) rate capability curves, and (**d**) retention rate curves of PVA–PANI HDGs with different PVA contents.

To evaluate the effects of the PVA content on the supercapacitor performance of PVA–PANI HDGs, CV, GCD, rate capability, and cycling stability tests were conducted (Figure 4). The CV curves of the coin cells were recorded in 1 M H_2_SO_4_ in the voltage range of 0–1.0 V at a scan rate of 20 mV·s^−1^ (Figure 4a). The CV profiles of the coin cell supercapacitors indicate excellent capacitive characteristics and rapid responses [7,8,9,19,20,21]. Among the samples containing different amounts of PVA, that with 5 wt.% PVA exhibited a larger CV area than those of 10 and 15 wt.% PVA. As the amounts of PVA decreased, the aspect ratio of the PANI nanomaterial increased; the 5 wt.% sample with a higher aspect ratio allowed greater current [21]. Therefore, the CV results proved the hypothesis that PVA–PANI HDG with a higher aspect ratio of PANI will store more electric charge.

To measure the capacitive performance of our coin cell samples employing PVA–PANI HDGs with different PVA contents, GCD curves were obtained at a current of 0.30 mA/cm^2^ in the voltage range of 0–1.0 V (Figure 4b). The symmetrical shape of the GCD curves indicates that reversible redox reactions of the PANI nanomaterials occurred within the HDGs. The IRs of the PVA–PANI HDGs with different PVA contents were measured from the voltage drop at the onset of the discharge curves [7,8,9,19,20,21]. The IRs (Ω/cm^2^) of the supercapacitors increased in the following order: 5 wt.% (29.6) < 10 wt.% (42.2) < 15 wt.% (121.4 Ω/cm^2^). The results mean that the growth of the PANI nanofibers was less limited by the smaller number of PVA chains, resulting in smaller voltage drops [21]. The smaller voltage drops and IRs are closely related to the enhanced electrical conductivity of the HDG with 5 wt.% PVA.

Based on the GCD analyses, the values of *C_A_*, *C_l_*, and *C_m_* of the HDGs with different PVA contents could be estimated, as shown in Figure 4c and Table 2. The side reactions inside coin cell supercapacitors increase with the current density, resulting in decreases in the specific capacitance of coin cell supercapacitors. The maximum value of *C_A_* of the 5 wt.% sample at a current density of 0.3 mA/cm^2^ was ~344.8 mF/cm^2^, which was larger than those of both the 10 wt.% (234.1 mF/cm^2^) and 15 wt.% (114.7 mF/cm^2^) samples. The same tendencies were observed for *C_l_* and *C_m_* (Figure 4c and Table 2). The *C_l_* values at a current density of 0.3 mA/cm^2^ increased in the following order: 15 wt.% (38.2) < 10 wt.% (78.0) < 5 wt.% (114.9 F/cm^3^). The values of *C_m_* at a current density of 0.3 mA/cm^2^ for the 5, 10, and 15 wt.% samples were 210.1, 142.6, and 69.9 F/g, respectively. The capacitance value obtained for the 5 wt.% sample (82.0% at a current density of 7.2 mA/cm^2^, as used for all three samples) decreased more slowly as the current density increased than that of the 10 wt.% sample (80.2%) and the 15 wt.% sample (77.5%; Figure 4c and Table 2). This suggests that the HDG with 5 wt.% PVA, which has a higher aspect ratio and a lower IR, is more suitable for preventing side reactions at higher currents [7,8,9,19,20,21]. The higher values of *C_A_* and *C_l_* for this HDG can facilitate the miniaturization of state-of-the-art supercapacitor devices.

To ensure the reliability of the coin cell supercapacitor devices containing PVA–PANI HDGs, the retention rates of the coin cells with different PVA contents were measured for 3000 cycles at a current density of 7.2 mA/cm^2^ (Figure 4d). The retention rates of the 5, 10, and 15 wt.% samples were reduced to 84.3, 80.6, and 78.2%, respectively (Table 2). Although capacitance losses are inevitable, volumetric expansion and chain scission of the PVA–PANI HDGs during the charge/discharge processes can be minimized by adding an optimal amount of PVA [4,21]. These results show that 5 wt.% was the optimal PVA amount for improving the rate capability and retention rate of the PVA–PANI HDGs.

**Figure 5:**
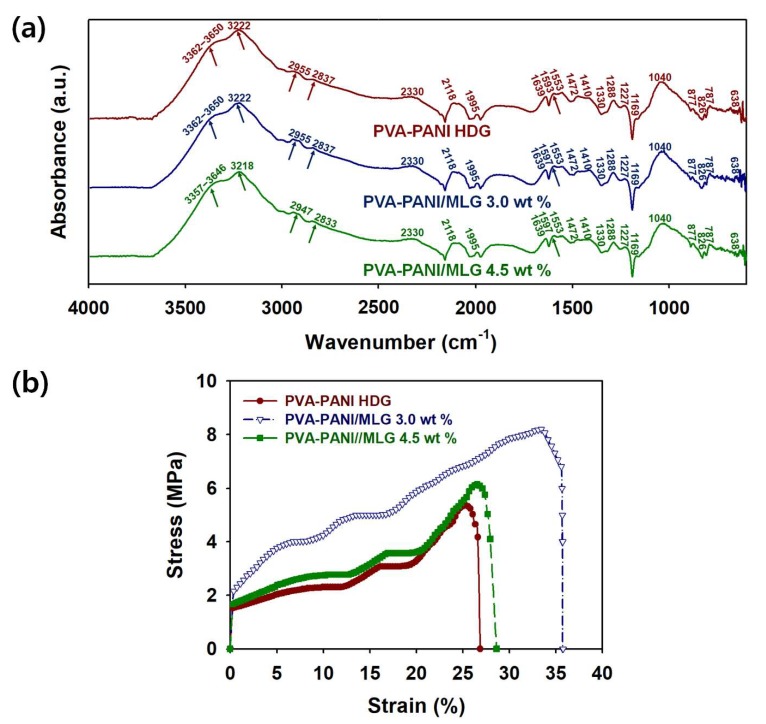
(**a**) FTIR spectra and (**b**) stress-strain (S-S) curves of PVA–PANI–MLG HDGs with different MLG contents: PVA–PANI (red), 3.0 wt.% MLG (blue), and 4.5 wt.% MLG (green). The concentration of PVA was fixed at 5 wt.%.

To confirm the bond structures of the PVA–PANI–MLG HDGs, the FTIR spectra of those with different MLG contents are shown in Figure 5a. The characteristic peaks for PANI are found at the following wavenumbers: 638, 787, 826, 877, 1040, 1227, 1169, 1288, 1472, 1553, 1593–1597, 1669, 1995, 2118, 2330, 2833–2837, 2947–2955, and 3218–3222 cm^−1^ (Table 3) [17,24,25]. The two distinctive bands found at approximately 1472 and 1593–1597 cm^−1^ originate from the C=C stretching of benzenoid rings (–N=B=N–) and quinoid rings (–N=Q=N–), respectively. The peak for the quinoid ring (1593 cm^−1^) shifted to a higher wavenumber (1597 cm^−1^) with the introduction of MLG. This redshift is indicative of extended conjugation paths via π–π stacking interactions between the MLG and PANIs [9,25]. The intensity ratio of the peaks for the quinoid (*I*_Q_) and benzenoid (*I*_B_) rings is associated with the protonation level of the PANI chains. The *I*_Q_/*I*_B_ ratio of the sample with 3 wt.% MLG (0.60) was significantly higher than that of the HDG without MLG (0.37) [25]. These results suggest that the MLG greatly increased electron delocalization and charge transport within the HDGs. After the addition of 4.5 wt.% MLG to the PVA–PANI structure, the *I*_Q_/*I*_B_ ratio of the sample (0.58) was lower than that of 3 wt.% MLG HDG. This indicates that excessive MLG clusters worsen the connectivity between the conductive areas within the HDG. Peaks for the B–O stretching vibrations of the –O–B–O– crosslinks are found at 1330 and 1410 cm^−1^, and peaks for the O–H stretching of boronic acid and PVA chains are observed at 3362–3650 cm^−1^ (Figure 5a and Table 3) [24]. This indicates that the –O–B–O– crosslinking between the PANI and PVA chains produced via the condensation reactions contributed significantly to the formation of the HDGs. After adding 4.5 wt.% MLG to the PVA–PANI chains, the band for the O–H stretching vibration shifted to a shorter wavenumber (3357–3646 cm^−1^) compared to that of 3 wt.% MLG (3362–3650 cm^−1^). These results imply that excessive MLG weakens the hydrogen bonding between the PVA and 3-ABA chains. These results suggest that the formation of HDGs is strongly influenced by the MLG content.

To confirm the effects of MLG on the mechanical properties of HDG, the stress–strain curves of PVA–PANI–MLG HDGs are shown in Figure 5b. The tensile strengths of the 3 wt.% MLG and PVA–PANI HDGs were 8.10 and 5.38 MPa, respectively (Table 4). Furthermore, the elongation at break of PVA–PANI HDG (26.88%) increased after adding 3.0 wt.% MLG (35.79%). Moreover, the Young’s moduli of the 3.0 wt.% MLG and PVA–PANI HDGs were 42.66 and 16.13 MPa, respectively (Table 4). However, the tensile strength (6.10 MPa), elongation at break (28.63%), and Young’s modulus (20.66 MPa) of 4.5 wt.% MLG HDG were lower than those of 3.0 wt.% MLG. These results indicate that the aggregation of MLG becomes significant above 3.0 wt.%, which reduces the strengthening effects of MLG on the mechanical properties of HDGs and hinders its formation. Therefore, the strengthening effect of MLG on HDG was optimized at an MLG content of 3.0 wt.%. Considering these results together, the optimum content of MLG is an important factor in producing strong and robust PVA–PANI–MLG HDG [4,17,18].

**Figure 6:**
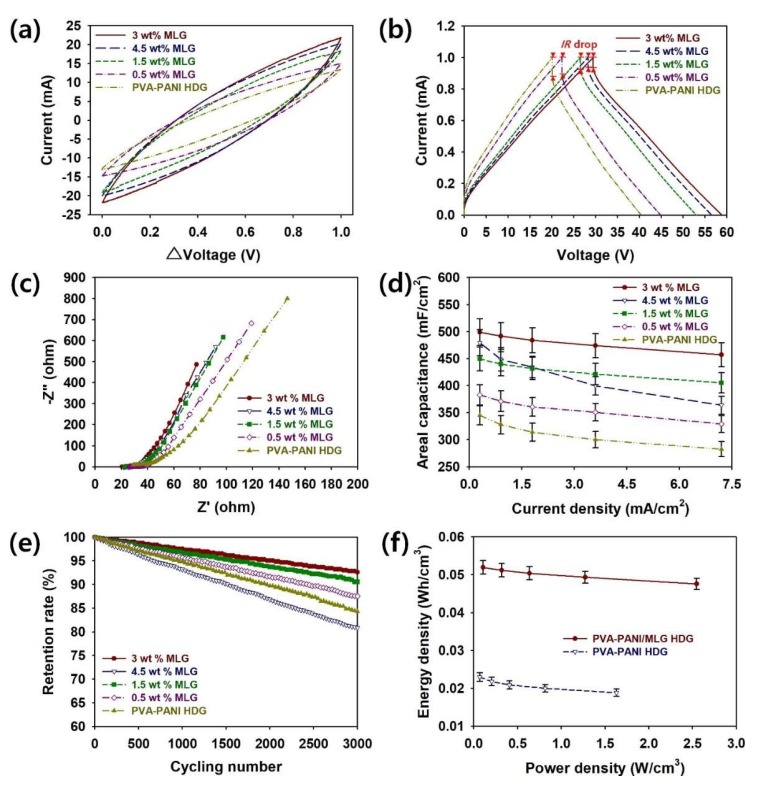
(**a**) CV curves, (**b**) GCD curves, (**c**) Nyquist plots, (**d**) rate capability curves, (**e**) cycling stability curves of PVA–PANI/MLG HDGs with different MLG contents: 3 wt.% (red), 4.5 wt.% (blue), 1.5 wt.% (green), 0.5 wt.% (purple), and 0 wt.% (yellow green). (**f**) Ragone plots for PVA–PANI–MLG and PVA–PANI HDGs. The concentration of PVA was fixed at 5 wt.%.

To evaluate the effects of the MLG content on the supercapacitor performance of PVA–PANI–MLG HDGs, CV, GCD, EIS, rate capability, cycling stability tests, and Ragone plots were utilized (Figure 6). The PVA content of the HDGs was fixed at 5 wt.%, as shown in Figure 3 and Figure 4. Figure 6a represents the CV curves of coin cells with different MLG contents. These CV curves were recorded in 1 M H_2_SO_4_ in the voltage range of 0–1.0 V at a scan rate of 20 mV·s^−1^. The rectangular shape of the curves indicates that MLG enhances capacitive behaviors and reduces response times [7,8,9,19,20,21]. Among the supercapacitors with different MLG contents, that with 3 wt.% MLG showed the largest CV area. Until the MLG content reached 3 wt.%, the current in the supercapacitors increased with the amount of MLG, whereas the current decreased after the addition of 4.5 wt.% or more MLG. Thus, the CV proved the hypothesis that the optimal amount of MLG significantly increased the electrochemical activity of the HDG [4,5,6,7,8,9].

Figure 6b shows the GCD curves of PVA–PANI–MLG HDG supercapacitors measured at a current of 0.30 mA/cm^2^ in the voltage range of 0–1.0 V. The IRs of the HDGs with different MLG contents were evaluated from the voltage drop at the onset observed in the discharge curves [7,8,9,19,20,21]. The IRs decreased in the following order: 0 wt.% (29.6) > 0.5 wt.% (24.0) > 1.5 wt.% (19.0) > 4.5 wt.% (17.4) > 3 wt.% (16.1 Ω/cm^2^). These results reconfirm that the π–π stacking interaction between MLG and PANI chains significantly enhanced the charge transport within the HDGs, resulting in smaller voltage drops [9,25]. However, the increased aggregation at an MLG content of 4.5 wt.% increased the IR of the HDG. Thus, the optimal MLG content was 3 wt.% for reducing voltage drops and IR [7,9,17,25].

To further investigate the effect of MLG on the electrochemical properties of the HDGs, Nyquist plots of the assembled samples employing PVA–PANI–MLG HDGs with different MLG contents were measured using EIS in the frequency range of 1–10 MHz (Figure 6c). Until the MLG content reached 3 wt.%, the sizes of the semicircles of the Nyquist plots decreased as the MLG content increased. This indicates that MLG enhances charge transport within the HDG, but undesirable impedances are caused by excessive MLG content. Accordingly, the HDG with 3 wt.% MLG exhibited the smallest charge transfer resistance. In the low-frequency region, vertical straight lines were observed in the Nyquist plot for each sample, indicating that the HDGs had effective ion diffusion and capacitive behaviors [1,19,20,21]. The equivalent series resistance of the PVA–PANI–MLG HDGs with different MLG contents increased in the order: 0 wt.% (28.8) > 0.5 wt.% (25.6) > 1.5 wt.% (22.5) > 4.5 wt.% (21.8) > 3 wt.% (20.5 Ω). At the optimal MLG content, it was evident that the interfacial resistance between the HDG structure and the electrolyte ions was decreased. The EIS results were consistent with the IRs observed in the GCD curves. The synergistic effects of PANI chains and MLG were advantageous for achieving HDG with superior electrical properties.

The values of *C_A_*, *C_l_*, and *C_m_* of the PVA–PANI–MLG HDGs with different MLG contents were estimated from the GCD analyses (Figure 6d and Table 5). The maximum value of *C_A_* (498.9 mF/cm^2^) was realized at an MLG content of 3 wt.%, which was superior to those of 4.5 wt.% (479.3), 1.5 wt.% (449.5), 0.5 wt.% (382.7), and 0 wt.% (344.8 mF/cm^2^) samples. The same trends were observed for *C_l_* and *C_m_* (Figure 6d and Table 5). The *C_l_* values at a current density of 0.3 mA/cm^2^ increased in the following order: 0 wt.% (114.9) < 0.5 wt.% (127.6) < 1.5 wt.% (149.8) < 4.5 wt.% (159.8) < 3.0 wt.% (166.3 F/cm^3^). The values of *C_m_* at a current density of 0.3 mA/cm^2^ for the 0, 0.5, 1.5, 4.5, and 3 wt.% samples were 210.1, 233.2, 273.9, 292.1, and 304.0 F/g, respectively. As the current density increased, the Faradaic reactions of HDGs became sluggish, and the effective thickness of the electrode material decreased [26,27]. Therefore, the capacitance of the HDG decreased as the current density increased. The capacitance value obtained from the 3.0 wt.% sample (91.6% at a current density of 7.2 mA/cm^2^, as used for all five samples) decreased more slowly as the current density increased than those of the 1.5 wt.% (90.1%), 0.5 wt.% (85.9%), 0 wt.% (82.0%), and 4.5 wt.% samples (76.0%; Figure 6d and Table 5). This implies that the MLG, which offers greater current within these devices, effectively retards the inevitable capacitance losses at higher currents, whereas the dramatic capacitance loss of the sample containing 4.5 wt.% MLG is associated with excessive aggregation of MLG [1,2,7,8,9,19,20,21]. This reaffirms that excessive aggregation of MLG limits the successful formation of HDG. Therefore, the optimal content of MLG to ensure the high rate capability of PVA–PANI–MLG HDG was found to be 3 wt.%.

To investigate the effects of MLG on the reliability of the PVA–PANI–MLG HDGs, the cycling stabilities of supercapacitors with different MLG contents are shown in Figure 6e. The cycling stability tests were conducted for 3000 cycles at a current density of 7.2 mA/cm^2^. The retention rates of the 4.5, 3.0, 1.5, 0.5, and 0 wt.% samples were reduced to 80.8, 92.6, 90.6, 87.6, and 84.3%, respectively (Table 5). These results confirm that the MLG effectively prevented the undesirable swelling and chain scission of the PVA–PANI chains during the repetitive charge/discharge processes [4,5,6,7,8,9]. When the MLG content reached 4.5 wt.%, a dramatic decrease in the retention rate was observed, mainly due to the deterioration of the HDG mechanical properties, as mentioned earlier (Figure 5b). Accordingly, it was evident that 3 wt.% was the optimal MLG content for improving the electrochemical activity, electrical conductivity, rate capability, and retention rate of the HDG.

To compare further the practical applicability of coin cells employing PVA–PANI–MLG and PVA–PANI HDGs, the Ragone plots (*P* versus *E*) of these materials are shown in Figure 6f. It was found that the supercapacitors with MLG could store more energy per unit volume than those without. The maximum values of *E* and *P* of the MLG HDG were 5.20 × 10^−2^ Wh/cm^3^ and 2.55 W/cm^3^, respectively, and these values were significantly superior than those of the HDG without MLG (2.30 × 10^−2^ Wh/cm^3^ and 1.63 W/cm^3^, respectively). Furthermore, the MLG HDG showed more gradual reductions in energy density than that without MLG. These results reaffirm that the synergistic effects of PANI chains and MLG were advantageous for combining the benefits from both the pseudocapacitor and EDLC mechanisms, resulting in remarkable improvements in both the energy density and power density of the coin cell supercapacitors [4,5,6,7,8,9].

The overall performances of state-of-art supercapacitors based on PANI and graphene are summarized in Table 6 [5,28,29,30,31,32]. Our work has shown higher or comparable performances compared to those of the previous work on PANI- and graphene-based supercapacitors. Especially, it was evident that the PVA–PANI–MLG HDG provides excellent capacity retention. This indicates that the high-performance coin cell supercapacitor was successfully realized by selecting PVA–PANI–MLG HDG as an electrode material.

## 4. Conclusions

In this comparative study, PVA–PANI–MLG composite HDGs with high scalability and remarkable mechanical, electrical, and electrochemical properties were demonstrated for use in a coin cell supercapacitor. The optimal amounts of PVA and MLG sheets to achieve the best performance were 5 and 3 wt.%, respectively. In FE-SEM images of these HDGs, HDG composed of PANI nanofibers could be achieved by choosing a PVA content of 5 wt.%, resulting in a higher aspect ratio for delocalizing electrons compared to those of the 10 and 15 wt.% samples. PVA–PANI HDG based on 5 wt.% PVA showed superior specific capacitance (344.8 F/cm^2^, 114.9 F/cm^3^, and 210.1 F/g) and retention rate (84.3% retention after 3000 cycles of GCD) than that with 10 wt.% PVA (114.7 F/cm^2^, 38.2 F/cm^3^, 69.9 F/g, and 80.6%). The FTIR spectrum of PVA–PANI–MLG HDG containing 3 wt.% MLG showed that it had successfully formed –O–B–O– bonds and enhanced conjugation paths. Due to the strong intermolecular forces between MLG, PANI, and PVA chains, remarkable tensile strength (8.10 MPa) and specific capacitances (498.9 F/cm^2^, 166.3 F/cm^3^, and 304.0 F/g) were realized by introducing 3 wt.% MLG into the PVA–PANI HDG. More importantly, remarkable retention rate (92.6% retention after 3000 cycles of GCD) and improved energy density (5.20 × 10^−2^ Wh/cm^3^) and power density (2.55 W/cm^3^) ensure the practical applicability of the HDG. Thus, our work on a coin cell supercapacitor based on PVA–PANI–MLG HDG will accelerate the application of this material as a promising power source for various mobile and miniaturized electronic devices.

## Figures and Tables

**Table 1:** Synthetic conditions of PVA-PANI HDGs with different sizes.

Size of Product		Solution A		Solution B		^4^ 3-ABA (mmol)	^4^ PVA (mL)	MLG Paste (mg)
^1^ Diameter (cm)	^1^ Height (cm)	^2^ APS (mmol)	^2^ DI Water (mL)	^3^ 5M HCl (mL)	^3^ Aniline (mmol)			
12.5	8.8	3.0	10	10	14.8	1.46	30	60–300
9.1	5.7	2.2	7.4	7.4	10.9	1.08	22	44–220
5	3.1	1.2	4.1	4.1	6.0	0.59	12	24–120

^1^ Size of products were estimated by measuring diameter and height of the obtained HDGs; ^2^ These reactants were used to produce solution A; ^3^ These reactants were used to produce solution B; ^4^ These reactants were used to copolymerize with aniline monomer.

**Table 2:** Supercapacitor performances based on PVA–PANI HDGs with different PVA contents.

PVA Content (%)	^1^ Areal Capacitance (*C_A_*, mF/cm^2^)	^1^ Volumetric Capacitance (*C_l_*, F/cm^3^)	^1^ Gravimetric Capacitance (*C_m_*, F/g)	^2^ Retention Rate at 7.2 mA/cm^2^ (%)	^3^ Retention Rate after 3000 Cycles (%)
5	344.8	114.9	210.1	82.0	84.3
10	234.1	78.0	142.6	80.2	80.6
15	114.7	38.2	69.9	77.5	78.2

^1^ These values were calculated according to GCD analyses. ^2^ These values were obtained from rate capability tests from different ranges of currents from 0.30 mA/cm^2^ to 7.2 mA/cm^2^. ^3^ These values were obtained from 3000 cycles of GCD tests.

**Table 3:** IR absorption bands of PVA–PANI–MLG HDGs.

^1,2^ Wavenumber (cm^−1^)	Assignments of Characteristic Bands
638	out-of-plane bending for NH_2_ of aromatic amine
787	out-of-plane bending for S=O of ammonium persulfate (APS)
826, 877	out-of-plane bending of *para*-substituted benzene ring
1040	symmetric stretching for S=O of ammonium persulfate (APS)
1227	C–N stretching of benzenoid amine
1288	C–N stretching of secondary aromatic amine
1330, 1410	B–O stretching of boronate crosslinks
1472	C=C stretching of benzenoid ring
1169, 1593–1597	C=C stretching of quinoid ring
1553	out-of-plane bending for N–H of secondary aromatic amine
1669	C=C stretching of aromatic ring
1995, 2118, 2330	out-of-plane bending for imine radical cation
2833–2837	C–H symmetric stretching
2947–2955	C–H asymmetric stretching
3218–3222	N–H stretching of boronic acid and PANI chains
3357–3646, 3362–3650	O–H stretching of boronic acid and PVA chains

^1^ These bands were found in FTIR spectra of PVA–PANI–MLG HDGs. ^2^ The concentration of PVA was fixed at 5 wt.%.

**Table 4:** Mechanical properties of PVA–PANI HDGs with different MLG content.

^1^ MLG Content (wt.%)	^2^ Ultimate Tensile Stress (MPa)	^2^ Elongation at a Break Point (%)	^3^ Young’s Moduli (MPa)
3.0	8.10	35.79	42.66
4.5	6.11	28.63	20.66
pristine PVA-PANI	5.38	26.88	16.13

^1^ The concentration of PVA was fixed at 5 wt.%. ^2^ These values were measured from stress-strain (S-S) curves of the samples during tensile tests. ^3^ Young’s moduli (E, MPa) was determined according to an equation E = stress (MPa)/strain (%).

**Table 5:** Supercapacitor performances based on PVA–PANI–MLG HDGs with different MLG contents.

^1^ MLG Content (%)	^2^ Areal Capacitance (*C_A_*, mF/cm^2^)	^2^ Volumetric Capacitance (*C_l_*, F/cm^3^)	^2^ Mass Capacitance (*C_m_*, F/g)	^3^ Retention Rate at 7.2 mA/cm^2^ (%)	^4^ Retention Rate after 3000 Cycles (%)
0	344.8	114.9	210.1	82.0	84.3
0.5	382.7	127.6	233.2	85.9	87.6
1.5	449.5	149.8	273.9	90.1	90.6
3.0	498.9	166.3	304.0	91.6	92.6
4.5	479.3	159.8	292.1	76.0	80.8

^1^ The concentration of PVA was fixed at 5 wt.%. ^2^ These values were calculated according to GCD analyses. ^3^ These values were obtained from rate capability tests from different ranges of currents from 0.30 mA/cm^2^ to 7.2 mA/cm^2^. ^4^ These values were obtained from 3000 cycles of GCD tests.

**Table 6:** Performance comparison of state-of-art supercapacitors based on PANI and graphene.

Electrode Material	Specific Capacitance	Cycling Stability (Cycles)	Tensile Strength	Ref.
PVA–PANI	306 F/cm^2^ 153 F/g	90% (1000)	5.3 MPa	5
N-doped graphene/PANI hydrogels	514.3 F/g	87.1% (1000)	-	28
PANI/rGO	423 F/g	75.0% (1000)	-	29
PANI/rGO/functionalized carbon cloth	0.47 F/cm^2^	75.5% (10,000)	-	30
PANI nanorod arrays/graphene	0.23 F/cm^2^	86.9% (8000)	-	31
rGO/Fe_3_O_4_/PANI composite	283.4 F/g	78.0% (5000)	-	32
PVA–PANI–MLG hydrogel	344.8 F/cm^2^ 234.1 mF/cm^3^ 210.1 F/g	92.6% (3000)	8.10 MPa	This work
